# Supplementary material for: Cell-specific roles for the conserved Galpha chaperone RIC-8 in cilia biology
Source: bioRxiv. 2026 Feb 9:2026.02.07.704597. Preprint. [Version 1] doi: 10.64898/2026.02.07.704597 (PMC12919077; doi:10.64898/2026.02.07.704597)
Supplement: Supplement 1 [file media-1.pdf]

Supplementary Figure 1. The RVxP motif precludes RIC-8 from localizing to the distal cilium. (A) Alignment of the *C. elegans* RIC-8 (NP 001023561) and human RIC8A (NP 068751) and RIC8B (NP 001317074) sequences showing presence of the RVIP motif (orange box) in the *C. elegans* protein. (B and C) Images (B) and quantification (C) of RIC-8^{WT}::TagRFP and RIC-8$^{\Delta RVIP}$::TagRFP localization in phasmid neurons of WT adults. Arrowheads: TZ; arrows: distal boundary of TagRFP signal. d: dendrite. Scale: 5 μm. * Different from wild type at $p<0.05$ (Mann-Whitney test).

Supplementary Figure 2. Ciliation is reduced in *RIC8A* and *RIC8B* KD RPE-1 cells. (A) Immunofluorescence images of fixed RPE-1 cells transfected with the indicated siRNAs and stained with anti-acetylated α-tubulin antibody and DAPI. siCTRL: non-targeting siRNA. Scale: 20 μm. (B) Quantification of ciliation in RPE-1 cells transfected with the indicated siRNAs. Each data point represents one KD experiment. *** Different from siCTRL at $p<0.001$ (Fisher's exact test).

A

Ce RIC-8	384	I------RVIPPLVSEEVQKRPEENNTLRG	407
Hs RICB8	386	D------QVLPPL--RDVTNRPEVGSTVRN	407
Hs RICBA	354	AQGWPPPQVLPPL--RDVRTRPEVGEMLRN	381

B

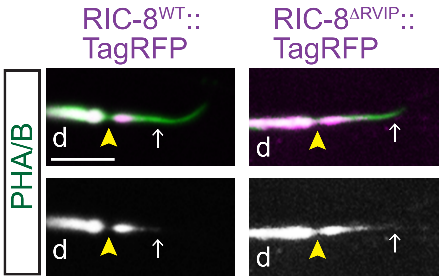

C

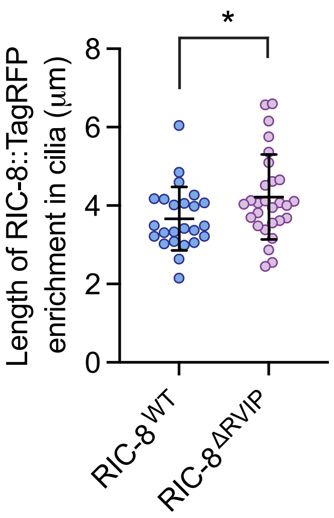

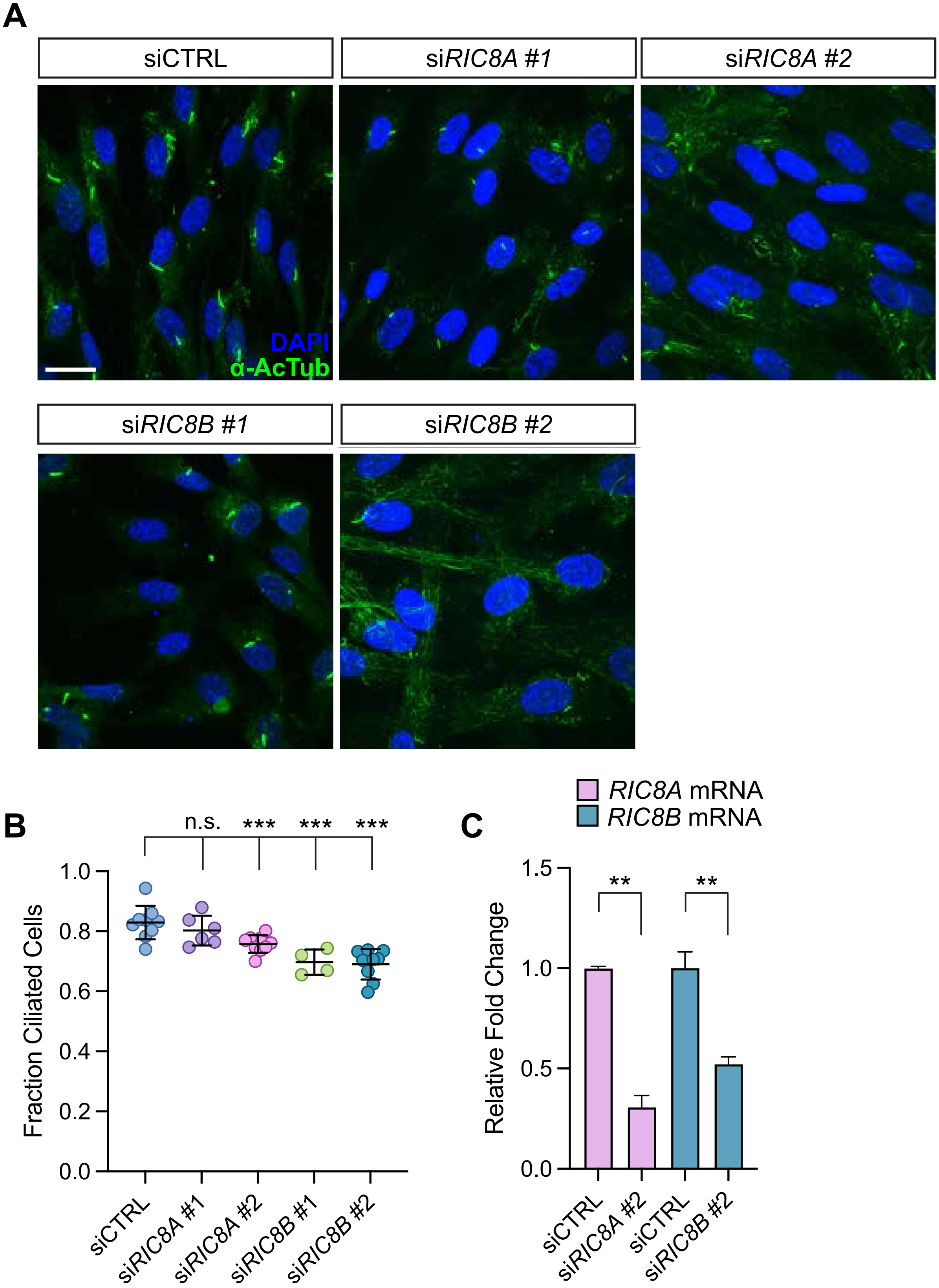
A
siCTRL
siRIC8A #1
siRIC8A #2
DAPI
α-AcTub
siRIC8B #1
siRIC8B #2
B
n.s.
***
***
***
Fraction Ciliated Cells
1.0
0.8
0.6
0.4
0.2
0.0
siCTRL
siRIC8A #1
siRIC8A #2
siRIC8B #1
siRIC8B #2
C
RIC8A mRNA
RIC8B mRNA
**
**
Relative Fold Change
1.5
1.0
0.5
0.0
siCTRL
siRIC8A #2
siCTRL
siRIC8B #2
